# Scaling up Tobacco Control in India: Comparing Smartphone to In-Person Training for Implementing an Evidence-Based Intervention to Reduce Tobacco Use Among Schoolteachers: Study Protocol

**DOI:** 10.1200/GO.22.59000

**Published:** 2022-05-05

**Authors:** Eve Nagler, Mangesh Pednekar, Daniel Gunderson, Glorian Sorensen, Prakash Gupta

**Affiliations:** ^1^ Dana-Farber Cancer Institute, Harvard T.H. Chan School of Public Health, Boston, MA; ^2^ Healis-Sekhsaria Institute for Public Health, Navi Mumbai, India; ^3^ Dana-Farber Cancer Institute, Boston, MA

## Abstract

Tobacco-related deaths are rising rapidly in low and middle-income countries (LMICs). In India, approximately 1.2 million people die each year from tobacco-related causes. Reducing tobacco-related deaths in LMICs will require large-scale implementation of evidence-based interventions (EBIs) that promote tobacco control. Currently, implementation of tobacco control EBIs in LMICs relies on in-person training, which has inherent logistical challenges that limit the ability to scale up these programs. New training models are needed to ensure tobacco control EBIs can be implemented broadly and at low cost, especially in rural and under-resourced areas where tobacco use is prevalent. In India, schoolteachers are respected community leaders and role models, representing an important channel for promoting tobacco control. We previously demonstrated the effectiveness of the Tobacco-Free Teachers, Tobacco-Free Society (TFT-TFS) program in increasing tobacco use cessation. To scale this EBI, we will compare two TFT-TFS-training models for headmasters—in-person versus smartphone-based—and examine the effect of each model on TFT-TFS program implementation and effectiveness.

**METHODS:**

Using a cluster-randomized design, we will randomize school headmasters in the Indian state of Madhya Pradesh to receive in-person or smartphone training. Once trained, headmasters in both groups will implement TFT-TFS within their schools. Accordingly, our aims are to (1) use a participatory, qualitative approach to develop the in-person and smartphone-based training models; (2) compare program implementation fidelity, effectiveness, and cost for both training models using process evaluation and survey data; and (3) identify factors affecting program implementation using mixed methods.

**RESULTS:**

This study will establish the effects of smartphone vs. in-person training on TFT-TFS implementation, effectiveness, and cost within schools in a low-resource setting.

**CONCLUSION:**

Our findings will provide insight into scaling up tobacco control EBIs in schools across India and other LMICs and inform the application of smartphone-based training for other public health-related EBIs in resource-constrained areas.

